# Burden of disease attributable to vitamin A deficiency in Iranian population aged less than five years: findings from the global burden of disease study 2010

**DOI:** 10.1186/s40200-017-0313-7

**Published:** 2017-08-09

**Authors:** Maryam Bahreynian, Mostafa Qorbani, Shohreh Naderimagham, Sara Nejatinamini, Asal Ataie- Jafari, Farshad Sharifi, Fahad Saqib, Alireza Khajavi, Morteza Mansourian, Ali Asghar Ahmadishokouh, Hamid Asayesh, Omid Safari, Roya Kelishadi

**Affiliations:** 1 Child Growth and Development Research Center, Research Institute for Primordial Prevention of Non-communicable Disease, Isfahan University of Medical Sciences, Hezarjerib Ave, Isfahan, Iran; 2 Non-Communicable Diseases Research Center, Alborz University of Medical Sciences, Baghestan Boulevard, Karaj, 31485/56 Iran; 3 Non-Communicable Diseases Research Center, Endocrinology and Metabolism Population Sciences Institute, Tehran University of Medical Sciences, Tehran, Iran; 4 Endocrinology and Metabolism Research Center, Endocrinology and Metabolism Research Institute, Tehran University of Medical Sciences, Tehran, Iran; 5 Department of Nutrition, Science and Research Branch, Islamic Azad University, Tehran, Iran; 6 Elderly Health Research Center, Endocrinology and Metabolism Population Sciences Institute, Tehran University of Medical Sciences, Tehran, Iran; 7 Health Management and Economics Research Center and Department of Health Education and Promotion, Iran University of Medical Sciences, Tehran, Iran; 8 Department of Applied Linguistics, Tarbiat Modares University, Tehran, Iran; 9 Department of Medical Emergencies, Qom University of Medical Sciences, Qom, Iran

**Keywords:** Global burden of disease (GBD), Vitamin a deficiency, Disability-adjusted life years, Years lived with disability, Iran

## Abstract

**Background:**

Vitamin A deficiency (VAD) is considered as one of the most serious public health concerns in developing countriesand the leading cause of mortality in under-five-year-old children.A large number of young children and pregnant women especially in low-income, non-industrialized communities are more susceptible to VAD. This study aims to report the burden of disease (BOD) attributable to VAD in Iranian population aged less than 5 years by using data of the Global Burden of Disease (GBD) study 2010.

**Methods:**

The GBD 2010 study calculated the proportion of deaths, years of life lost (YLLs), and years lived with disability (YLDs) and disability-adjusted life years (DALYs) attributable to VAD by using the comparative risk assessment (CRA). VAD defined as low serum retinol concentrations (plasma retinol concentration < 0.70 umole/L) among children aged less than five. The VAD outcomes consisted of mortality due to diarrhea, measles, malaria, neglected infectious diseases, morbidity due to malaria (children < 5 years), low birth weight and other perinatal conditions. Uncertainty in the estimates is presented as 95% uncertainty interval (UI).

**Results:**

In 1990, there were 371 (95% UI: 166,665) DALYs due to VAD per 100,000 under five-year-old Iranian children in both sexes. The DALYs rate had a downward trend throughout the following years and reached to 76 (95% UI: 33–139)in 2010.The DALYs in children aged under 5 years was 378 (95% UI: 153–747) years for boys and 363 (95% UI: 148–692) years for girls in 1990 which fell to 79 (95% UI: 32–149) and 73 (95% UI: 29–138) in boys and girls in 2010, respectively. The rates of YLDs attributable to VAD changed in both sexes from 87(95% UI: 34–162) in 1990 to 46 (95% UI: 17–69) in 2010. The highest rate of YLDs attributed to VAD was observed in children aged 1–4. On the other hand, the YLLs were mostly in the 0–1-year-oldchildren in all years except 2010.

**Conclusion:**

It was found that DALYs attributable to VAD in 1990, followed by a considerable reduction rate after a period of two decades, in 2010. Additional studies on the burden of diseases particularly at sub-national level with more accurate data are recommended.

## Background

Vitamin A is an essential micronutrient for growth, cell differentiation and proliferation, reproduction, eyesight, improved immune system, maintenance and protection of membrane integrity [1].Vitamin A and its active metabolite form, namely retinoic acidare essential for the development and function of different tissues such as the immune system [1].

Poor nutritional status, inadequate intake of vitamin A and infections such as diarrhea and measles are the most common causes leading to vitamin A deficiency (VAD) [2]. VAD is characterized using a serum or plasma retinol concentration of <0.7 μmol/L as cut-off for VAD [1, 2].

VAD is recognized as a serious public health problem in developing countries [3–5]. A large number of young children and pregnant women especially inlow-income and non-industrialized communities are more susceptible toVAD. It results in night blindness, xerophthalmia, infection, iron deficiency anemia, and increased mortalityrate [2].According to the World Health Report (2011), the global prevalence of VAD in 0–4- year- old children was close to 21%, and the frequency of nightblindness was estimated to be 5% among pregnant women with the greatest prevalence in Asian and African countries [6–8].It is also estimated that approximately 190 million preschool children and19 million pregnant women are affected by VAD, worldwide [6]. VAD is a major cause of mortality in children under-five. Any improvement in vitamin A status might promote resistance to its health consequences and reduced mortality rate up to 23% [9].

Furthermore, VAD mightlead to the burden of diseases (BOD) as increasing the risk of susceptibility to infection, birth defects, blindness, cognitive disorders and premature mortality rates [2, 5, 10–12]. Previous global risk factor assessment revealed that 0.8 million (1.4%) of deaths worldwide are due toVAD [13],andVAD was responsible for 1.8% of the global burden of diseases (GBD) measured in disability-adjusted life years (DALYs) [7, 14].GBD is a study conducted by the Institute for Health Metrics and Evaluation (IHME) that calculated the GBD in 2010 [15].

Little information is available regarding vitamin A status among Iranian individuals [12] and there is no report of measured BOD related to VAD in Iran. This study aims to report the BOD attributable to VAD in Iranian population aged less than 5 years by using data of the GBD study from 1990 to 2010 [15].Furthermore, this study aims to compare the results with the similar findings and discuss about the limitations of the GBD project for estimating burden of VAD.

## Methods

The GBD 2010 study calculated the proportion of death, years of life lost (YLLs), years lived with disability (YLDs), and DALYs attributable to VAD between 1990 and 2010. The GBD study 2010 has calculated the proportion of death and DALYs attributable to VAD between 1990 and 2010. The details of data, data quality, and statistical models for GBD Study 2010 estimation are described previously [16–22]. The GBD 2010 study has categorized this nutrient deficiency as a risk factor in the cluster “childhood and maternal under nutrition” [23]. VAD was defined as low serum retinol concentrations (<0.70 μmol/L) among children under-5 years of age. The outcomes resulting from VAD were considered as mortality due to diarrhea, measles, malaria, miscellaneous infectious causes of disease, morbidity due to malaria (children <5 yr) as well as low birth weight and other prenatal conditions.

The burden was estimated for all ages and in both sexes. However, in this study, the measures for under-five-year-old age group are reported becausethe burden of VAD was not noticeable in the other age groups. Details about estimating disease burden attributable to each risk factor have been explained elsewhere [23].After selecting risk-outcome pairs, distribution of exposure to each risk factor was estimated according to published and unpublished data sources. For vitamin A, key sources were comprehensive review of data from multiple sourcesincluding the World Health Organization (WHO) and Micronutrient Initiative summary reports, journal articles, published and unpublished survey reports.

In the last step, attributable deaths or DALYs to VAD were calculated by comparing the present distribution of exposure with the theoretical-minimum-risk counterfactual exposure distribution for each age group, sex, and year (1990–2010). For VAD, theoretical-minimum-risk exposure distribution was considered as 100% of the population being without VAD.

For each risk factor and disease pair, population attributablefraction (PAF) was calculated in each age and sex group. Uncertainty in the relative risks, exposure estimates, theoretical minimum risk distributions and uncertainty inthe background outcome rates have been propagated into the final estimates. Uncertainty in the estimates is presented as 95% uncertainty interval (UI).

## Results

**Fig. 1:**
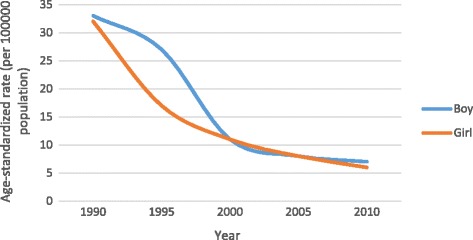
Age-standardized DALYs rate (per 100000 population) to vitamin A deficiency by sex and year in Iran

The attributable burden of VAD among under-5-year-old age group during 1990–2010 is presented in Table 1. There were 371 (95% UI: 166–665) DALYs due to VAD per 100,000 Iranian children in both sexes. The DALYs rate had a downward trend throughout the following years and reached to 76 (95% UI: 33–139) in 2010. The burden of DALYs in children aged under 5 years was 378 (153–747) years for boys and 363 (95% UI: 148–692) years for girls in 1990 which fell to 79 (95% UI: 32–149) and 73 (95% UI: 29–138) respectively in boys and girls in 2010. Fig. 1 shows age-standardized DALYs rate (per 100,000 population) attributable to VAD by sex and year. The age-standardized DALYs rate had a downward trend throughout the following years in boys and girls and reached to 7 [95% UI: 3, 13] and 6 (95% UI: 3, 12) in 2010 in boys and girls respectively.

According to the GBD data, VAD is a risk factor for nutritional deficiencies and infectious diseases including diarrhea, lower respiratory infections, meningitis, and other common infectious diseases. However, the majority of DALYs attributable to this risk factor were from infectious diseases and nutritional deficiencies had a negligible role (data are not shown in table).

**Table 1:** Disability-adjusted life-years attributable rate (per 100000 population) to vitamin A deficiency by age, sex and year in Iran

	DALY [95% UI]				
Both sexes	1990	1995	2000	2005	2010
0-1 year	374[111,878]	183[58,419]	94[29,201]	63[18,139]	41[14,93]
1-4 year	377[170,682]	203[94,353]	133[56,232]	98[39,179]	86[36,160]
Under 5 year	371[166,665]	196[90,351]	124[52,217]	90[35,163]	76[33,139]
Age-standardized	33[15,59]	17[8,31]	11[5,19]	8[3,14]	7[3,12]
Boy					
0-1 year	423[107,1060]	204[54,537]	102[28,262]	66[16,168]	44[12,119]
1-4 year	375[152,758]	205[87,397]	135[53,263]	101[37,194]	89[36,168]
Under 5 year	378[153,747]	202[83,370]	128[50,242]	93[34,176]	79[32,149]
Age-standardized	33[14,66]	27[11,49]	11[4,21]	8[3,16]	7[3,13]
Girl					
0-1 year	324[72,865]	162[37,461]	86[21,244]	59[13,158]	38[10,110]
1-4 year	378[155,712]	200[85,359]	130[54,234]	95[37,181]	83[33,158]
Under 5 year	363[148,692]	191[82,341]	120[49,216]	86[33,161]	73[29,138]
Age-standardized	32[13,61]	17[7,30]	11[4,19]	8[3,14]	6[3,12]

*DALY* Disability-adjusted life-years; *UI* Uncertainty interval

Tables 2 and 3 show the changes in YLDs and YLLs in children less than 5 years in Iran between 1990 and 2010.The rates of YLDs attributable to VAD and in both sexes changed from 87(95% UI: 34–162) in 1990 to 46 (95% UI: 17–69) in 2010.VADcaused the most disability burden (YLD) in the 1–4 year age group compared to children aged less than 1 year. On the other hand, burden of death affected mostly the 0–1- year age group in all years except for 2010 (Table 3).

**Table 2:** Years lived with disability attributable rate (per 100000 population) to vitamin A deficiency by age, sex and year in Iran

	YLD [95% UI]				
Both sexes	1990	1995	2000	2005	2010
0-1 year	18[6,43]	16[5,37]	15[5,34]	12[4,30]	11[3,25]
1-4 year	104[40,193]	81[32,151]	73[28,141]	59[21,116]	55[21,107]
Under 5 year	87[34,162]	69[27,129]	61[24,119]	50[18,97]	46[17,89]
Age-standardized	8[3,14]	6[2,11]	5[2,10]	4[2,9]	4[2,8]
Boys					
0-1 year	20[5,52]	18[5,46]	16[4,40]	14[3,36]	12 [3,31]
1-4 year	101[37,197]	80[30,156]	72[27,1437]	59[20,122]	55[20,112]
Under 5 year	85[31,164]	68[25,132]	61[22,121]	50 [17,101]	46[17,93]
Age-standardized	7[3,14]	6[2,11]	5[2,10]	4[1,9]	4[1,8]
Girls					
0-1 year	16[4,43]	14[3,37]	13[3,34]	11[3,29]	10[2,28]
1-4 year	106[38,206]	83[30,159]	73[27,140]	59[20,118]	54[19,114]
Under 5 year	88[32,171]	70[25,132]	62[23,118]	49[17,98]	45[16,93]
Age-standardized	8[3,15]	6[2,11]	5[2,10]	4[1,9]	4[1,8]

*YLD* Years lived with disability; *UI* Uncertainty interval

**Table 3:** Years of life lost attributable rate (per 100000 population) to vitamin A deficiency by age, sex and year in Iran

	YLL [95% UI]				
Both sexes	1990	1995	2000	2005	2010
0-1year	356[106,839]	167[52,391]	80[24,173]	50[14,115]	31[9,74]
1-4 year	273[113,537]	121[51,229]	60[26,112]	38[14,75]	31[12,63]
Under 5 year	284[116,545]	127[52,239]	63[26,114]	40[15,75]	31[12,58]
Age-standardized	25[10,48]	11[5,21]	6[2,10]	4[1,7]	3[1,5]
Boys					
0-1 year	403[100,1016]	186[48,501]	86[23,231]	53[11,139]	32[8,94]
1-4 year	274[98,573]	124[47,268]	64[23,123]	42[14,91]	34[11,72]
Under 5 year	293[108,622]	133[52,268]	66[24,127]	43[15,91]	33[12,68]
Age-standardized	26[10,55]	12[5,24]	6[2,11]	4[1,8]	3[1,6]
Girls					
0-1 year	308[66,831]	148[33,425]	73[17,217]	48[10,136]	29[6,86]
1-4 year	272[103,540]	118[47,230]	57[22,113]	35[12,75]	28[10,60]
Under 5 year	275[104,561]	121[48,238]	59[23,119]	37[13,76]	28[10,59]
Age-standardized	45[17,92]	11[4,21]	5[2,11]	3[1,7]	2[1,5]

*YLL* Years of life lost; *UI* Uncertainty interval

The ranking of VAD as a risk factor in children less than 5 years between 1990 – 2010 are shown in Table 4. The ranking dropped from the eighth leading risk factor in 1990 to the 9th in 2010 in the 0–1 year group, and from 6th to 7th in the 1–4 year age group.

**Table 4:** Vitamin A deficiency mean rank [95% UI] for both sexes in 1990 and 2010

DALY	1990 mean rank (95% UI)	2010 mean rank(95% UI)
0-1 year	8.8 (7-10)	9.7 (7-11)
1-4 year	6.2 (3-9)	7.3 (4-10)
Under 5 years	8.6 (7-11)	8.7 (7-11)
Age standardized	18.9 (17-21)	23.5 (20-25)

*DALY* Disability-adjusted life-years; *UI* Uncertainty interval

The disease burden attributable to VAD decreased from 0.15% (95% UI: 0.07–0.78) of total DALYs in 1990 to 0.02% (95% UI: 0.01–0.04) in 2010.

## Discussion

The current study represents the first attempt to measure the disease burden attributable to VAD during 1990 – 2010 in Iran by using the data of the GBD study 2010 measured in DALYs. According to these findings, higher DALYs related to VAD was observed in the year 1990, followed by a considerable reduction rate after a period of two decades in 2010. The burden of VAD among Iranian children under 5 was estimated to be 371 and 76 DALYs per 100,000 populations in 1990 and 2010, respectively.

VAD is a major public health issue among preschool children living in developing countries and contributes to higher rates of infants and/or child mortality and disease burden compared to developed ones. The highest VAD prevalence has also been found in Africa, Mali, Ethiopia, Nigeria and Egypt. Higher incidence of VAD is almost occurred in young children and pregnant women of low income and non-industrialized countries [3, 11, 24–27]. Previous reports have demonstrated that 33.8% of 0–4 year old South-African children are VAD, and was responsible for 28% of all deaths resulted from diarrheal disease, 23% from measles and 21% from malaria among them in the age group of 0–4 years. Improved vitamin A status could prevent 1.3–2.5 million of almost 8 million late infancy and pre-school aged deaths occurring each year in developing countries [7].

During the past two decades, an outstanding improvement in health and sanitation combined with greater availability of health service facilities as well as improvement in the health knowledge of the population contributed in the control of preventable communicable diseases in Iran. However, a shift in the disease burden toward non-communicable chronic diseases (NCDs) occurred [28].Similar patterns of increasing trend in the prevalence of NCDs have also been emerged in other parts of the world [29–31].Likewise, the decreasing trend in the GBD related to VAD during the two recent decades was similar to the global reports as well as Middle-Eastern countries such as Iraq and Turkey. Declining the VAD prevalence might be due to widespread vitamin A supplementation with measles immunization among at risk population [32]. It seems that unfavorable lifestyle changes along with industrialization and urbanization increased the degree of exposure to NCDs risk factors as a major health concern. The worldwide rising epidemic of obesity-related diseases reflects the significant alterations in society and behavioral patterns of community undergoing the nutritional and epidemiological transition over recent decades [33, 34].

In the current study, higher burden of VAD was observed among infants aged 0–1 year during 1990s. VAD in infancy is an indicator of inadequate vitamin A stores during pregnancy. As was found by most studies from developing countries, VAD pregnant mothers are more likely to have infants with insufficient vitamin A stores [35, 36].It seems that the deficiency in vitamin A status could remain even in the period of lactation [37].However, because of its vitamin A content, human milk is suggested to have a protective role against VAD [38].Previous reports have revealed that breastfed infants were more likely to have sufficient vitamin A levels and subsequently reduced frequency of infections [5, 39].

Over the past two decades, economic growth, urbanization, and the profound changes in social levels and the economic situation of the families have led to the improvement in vitamin A status of children [5].Additionally, higher levels of parental education and nutrition knowledge might be another reason associated with greater attention to healthy eating patterns, including vitamin A rich products [40].These factors might interfere with the results of the present study.

Another reason for higher disease burden of VAD during 1990s could be the establishment and availability of health care centers as well as health care facilities across the country. Over the past two decades, the number of active Health Houses and health centers have increased considerably proportional to the population size [41].

It is important to highlight that VAD is not a direct contributor to death by itself. The main determinant of higher mortality rates among children of developing countries would be the widespread prevalence of infections as diarrhea, respiratory disease, and measles [5, 42]. VAD might exacerbate the incidence, duration, and severity of infections through its immunomodulatory effects [43, 44].Promoting intervention-treatment programs to reduce the incidence of infectious diseases and improve child survival could help to explain the decrease in the VAD burdenin Iran and across the world.

Despite the equally available centers offering health care, utilization of health services in some provinces like Sistan and Baluchestan, it is estimated to be low compared to the rest of the country. It seems that factors such as people^’^s attitude might play a role in this regard [45].Furthermore, lack of sufficient nutrition knowledge, high cost, and seasonal variations in vitamin A containing foods could be other barriers to modify overall diet quality in low-income households [5, 46, 47].

In this regard, it is worth mentioning that VAD had large variations within different rural areas and provinces in Iran. Therefore, providing an estimation of aggregate data on VAD mortality for all provinces of the country could conflict the results [42]but the GBD data are more accurate at global and national levels. In addition, GBD estimations aremore model-driven than data-driven for adopting appropriate policies to promote the health of the regions of a country by the authorities of health section. Therefore, sub-national studies with more data are necessary to estimate the burden of diseases more accurately to provide evidence for health policy makers. In this regard, recently, National and Sub-national Burden of Disease (NASBOD) study is conducted in Iran to calculate the burden of diseases, injuries and risk factors at national and sub-national levels from 1990 to 2013 [48, 49].Estimation of prevalence and burden of nutritional diseases and risk factors at sub-national level is a sub- project of NASBOD study [50].The above-mentioned study is a valuable one that benefits all published and unpublished data in the country and two statistical methods [51].

Unlike the aforementioned limitations, exploring and describing the burden of diseases attributable to VAD could potentially provide useful information on the importance of the problem. For instance, from 1 in 7 to 1 in 3 deaths might be prevented through improvement of nutritional adequacy of children aged 6 months up to preschool age in the developing world [35].Additionally, health system development, public health successes are areas where the country would need urgent action.

## Conclusion

We found a considerable declining trend in the BODattributable to VAD among Iranian pre-school children in a period of 20 years. Additional studies on the burden of diseases including visual impairment and skin problems due to VAD are recommended.

## Abbreviations

BOD: Burden of disease
CRA: Comparative risk assessment
DALYs: Disability-adjusted life years
UI: Uncertainty interval
VAD: Vitamin A deficiency
YLDs: Years lived with disability
YLLs: Years of life lost
